# A Systematic Analysis of the Available Human Clinical Studies of Dental Implant Failure in Patients with Inflammatory Bowel Disease

**DOI:** 10.3390/medicina58030343

**Published:** 2022-02-24

**Authors:** Andrada Voina-Tonea, Anca Labunet, Adriana Objelean, Florin Onisor, Simion Bran, Alexandru Mester, Andra Piciu, Sorina Sava

**Affiliations:** 1 Department of Dental Materials, Iuliu Hațieganu University of Medicine and Pharmacy, 400012 Cluj-Napoca, Romania; andrada.tonea@umfcluj.ro (A.V.-T.); labunet@yahoo.com (A.L.); adriana.caracostea@umfcluj.ro (A.O.); savasorina@yahoo.com (S.S.); 2 Department of Maxillofacial Surgery and Implantology, Iuliu Hațieganu University of Medicine and Pharmacy, 400012 Cluj-Napoca, Romania; dr_brans@umfcluj.ro; 3 Department of Oral Health, Iuliu Hațieganu University of Medicine and Pharmacy, 400012 Cluj-Napoca, Romania; 4 Department of Medical Oncology, Iuliu Hațieganu University of Medicine and Pharmacy, 400012 Cluj-Napoca, Romania; andra.piciu@umfcluj.ro

**Keywords:** inflammatory bowel disease, Crohn’s disease, ulcerative colitis, dental implant, implant therapy

## Abstract

*Background and objectives:* The aim was to evaluate the current literature on the influence of inflammatory bowel disease (ulcerative colitis/Crohn’s disease) in dental implant osseointegration in human clinical studies. *Materials and methods:* This review was conducted under the Preferred Reporting Items for Systematic Review and Meta-Analysis guidelines. PubMed, Scopus, and Web of Science databases were electronic screened to find relevant articles published until October 2021. The inclusion criteria consisted of human clinical studies that reported the use of dental implant in patients diagnosed with inflammatory bowel disease. Risk of bias was assessed according to The Strengthening the Reporting of Observational studies in Epidemiology criteria. *Results:* A total of 786 studies were identified from databases. Of these, six studies were included in the review and reported the use of implants in patients with Crohn’s disease. No articles were available for ulcerative colitis. Included articles indicated that Crohn’s disease may determine early and late implant failure. Besides Crohn’s disease, several patients presented associated risk factors and systemic disease that determined implant failure. *Conclusions:* The presence of clinical studies on the influence of IBD in implant therapy is low. When recommending an implant therapy to IBD patients, the multidisciplinary team should be aware of side effects and a close collaboration between members of this team is necessary. More data are needed to sustain the effect of IBD on implant therapy.

## 1. Introduction

The main pathologies belonging to the set of inflammatory bowel diseases (IBD) are defined as ulcerative colitis (UC) and Crohn’s disease (CD) [1]. Pathogenetic mechanisms are represented by the imbalance in the community of microorganisms, caused by the synergic effect of environment related factors and genetic predisposition [1]. UC and CD manifest repercussions in the mediation of the immune response, bacterial destruction, and function of the intestinal barrier [1,2]. As a consequence, in the context of IBD, the abnormal balance of bacterial and fungal colonies produces an elevated immune response, with increased permeability of the mucosal layer and malfunction of the epithelium [1,2].

These two pathologies are characterized by intestinal, but also by extra-intestinal manifestations [3]. The recurring aspect of clinical inflammatory episodes, together with intestinal blockage, loose stools, associated with mucosal and/or blood discharge, febrile events and abdomen aches outline the picture of IBD symptomatology [3]. Extra-intestinal expressions of IBD are related to the muscular system, pulmonary system, renal and ocular manifestations, and dermatological consequences [4]. 

A challenge in the daily practice of physicians is represented by the identification of oral manifestations of IBD. The oral signs of the pathology are characterized by an increased complexity and variety of expression, which implies a high difficulty in their identification [4]. Oral manifestations can be characteristic for the disease, but can also occur due to the deficit of absorption or as a result of the undertaken medical treatment [3,4,5]. They can appear before establishing the certainty of IBD or can coexist with it and also interact with its medical treatment [4]. Inflammatory stomatitis is more often linked to UC, while aphthae, inflammation of the oral mucosa and mucosal lessons, and ulcerations appear more frequently in the context of CD [5,6]. Special attention should be offered to the identification of orofacial granulomatosis in pediatric patients. This uncommon pathology can represent an important sign of CD in young persons or even guide the practitioner towards other significant systemic pathologies. Other oral manifestations can occur in the form of several carious processes or periodontal pathologies [5,6]. 

In the field of dentistry, the presence of IBD may change the course of treatment, in cases where the insertion of dental implants is necessary. In this context, osteoporosis and systemic osseous destructions can be observed, with an elevated osseous turnover and a low bone development rate [6]. All these aspects have an important implication in the process of osseointegration of dental implants [6]. Furthermore, the lack of nutrition in people suffering from IBD and the occurrence of autoimmune inflammatory processes at the site of bone and implant junction, along with other processes in various parts of the organism, may lead to insufficient osseous formation around the implant [6,7]. The risk of failure and the poor implant prognosis of patients with IBD is commonly acknowledged by medical specialists, knowing that other systemic disorders also have the capacity to induce the same undesirable consequences [7,8,9]. Another aspect that should be taken into account is the peri-implant bone loss. Sinjari and coworkers, observed in their clinical trial that bone resorption may appear during the placement of healing abutment. This bone loss may be present in the first year of loading due to the presence of a microgap between fixture and abutment. In this area, a bacterial colonization initiates an inflammation, which, if not treated, may transform into peri-implant bone resorption [10]. In another study, D’Ercole and coworkers indicated that microbial contamination may compromise implant stability [11]. During bacterial inoculation, peri-implant tissues are compromised if any appropriate care is not initiated [10,11].

Given the high significance of oral manifestations in the context of IBD and the difficulty of their identification, the close collaboration between medical specialties, including dental professionals, is of very high importance [6,7]. Oral surgeons should take these types of pathologies into consideration and perform their treatment with special care, regarding the osseointegration and the evolution of the healing process, not overlooking the signs of implant failure that may occur in this type of cases. In regards to the last statement, the aim of this review was to systematically evaluate the current electronic literature regarding dental implant failure in IBD (UC/CD) in human clinical studies.

## 2. Materials and Methods

This review was conducted under the PRISMA (Preferred Reporting Items for Systematic Review and Meta-Analysis) guidelines stated by Moher et al. [12]. The focused question of this study was developed under PICOS (Patient, Intervention, Comparison, Outcome, Study design) criteria: “Are patients diagnosed with inflammatory bowel disease (Crohn’s disease/ulcerative colitis) (P) with dental implants rehabilitation (I) compared to systemic healthy patients (C), to present early/late implant loss (O) in human clinical studies (S)?”.

### 2.1. Inclusion and Exclusion Criteria

All the included studies needed to follow the inclusion criteria: human clinical studies (clinical trials, prospective studies, retrospective studies, case series, and case reports), articles published in English language, patients diagnosed with IBD (CD or UC), and treatment with dental implants (failure/successful implant osseointegration). Exclusion criteria consisted of: reviews, letters to the editor, experts’ opinion, conference paper, comments, in vitro/animal studies, patients with other diagnosis of IBD, no reports of implant therapy, and incomplete/unpublished data.

### 2.2. Literature Search

Three databases (PubMed, Scopus, and Web of Science) were electronic screened to find relevant articles published from the date of inception until October 2021). A systematic search was also performed in the grey literature in ClinicalTrial.gov and OpenGrey. The search strategy consisted of three steps. The first step consisted in title screening by two independent reviewers in order to exclude irrelevant papers. Then, in the second step, abstracts were assessed in order to correspond with the question of our review. In the last step, full-text articles previously obtained were assessed in order to correspond with inclusion criteria. If any disagreement was present regarding inclusion, a third reviewer was involved to resolve the issue. The level of agreement between reviewers was established using Cohen’s kappa index [13]. The electronic search on the selected databases was conducted using the following search strategy: (“inflammatory bowel disease” OR “IBD” OR “crohn disease” OR “ulcerative colitis”) AND (“dental implant” OR “oral implant” OR “implant therapy” OR “endoosseous implant” OR “osseointegrated implant” OR “dental implant loss” OR “implant loss” OR “dental implant failure” OR “implant failure” OR “titanium implant” OR “zirconia implant” OR “Ti implant” OR “Zr implant”).

### 2.3. Data Extraction

The following data were considered using a standard data collection form: first author, year of study, country, type of study, sample size, type, and diagnosis of IBD, characteristics of dental implant, results before and after the implant treatment, and conclusions.

### 2.4. Risk of Bias

Risk of bias was determined using The STROBE statement (The Strengthening the Reporting of Observational studies in Epidemiology) published by Vandenbroucke et al. [14]. STROBE assessment of each included study is analyzed by two reviewers and in case of disagreements, a third one is involved. STROBE consists in 7 criteria in which is evaluated the study design, types of participants, sample size, presence of any variables, potential confounders outcomes, and if statistical analysis was performed. Each of these 7 criteria is quantified with “1” (if criteria is stated in the included article) or “0” (if it is not present). Maximum score possible is 7; after the final score is summed, quality of the study assessed is included in one of the three thresholds available: low (score 0–3), acceptable (score 4–5), or high (score 6–7). 

### 2.5. Statistical Analysis

The main goal of our study was to obtain a meta-analysis, but due to heterogeneity of patients’ characteristics and assessment of implant therapy, statistical analysis could not be made.

## 3. Results

### 3.1. Search Results

**Figure 1:**
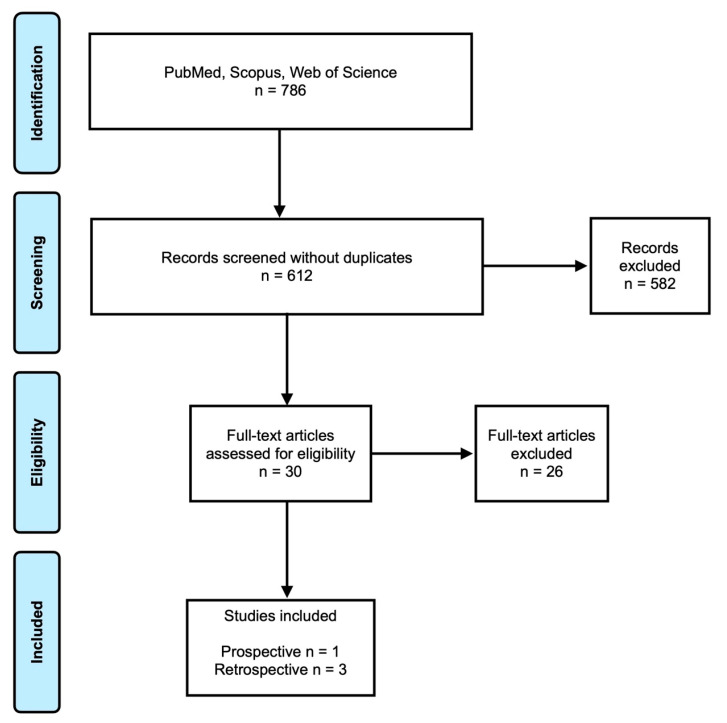
PRISMA flow chart.

The initial electronic search from the three databases, a total of 786 studies were identified (Figure 1). The grey literature in ClinicalTrial.gov and OpenGrey, showed no relevant results. After excluding the duplicates, 612 studies were assessed for eligibility. Then, the articles were reviewed by title and abstract. Of these, 30 studies were full-text assessed for eligibility. In the end, four clinical studies were included in this review. 

### 3.2. General Characteristics

The retrieved studies were published between 2002 and 2015. Regarding the type of studies, one was a prospective study [15] and three were retrospective studies [16,17,18]. The studies were conducted in Belgium [15,16,17,18], USA [19], and Italy [7]. The sample sizes of the clinical studies varied between studies; two studies did not report the number of IBD patients and only two studies [15,18] mentioned the number of IBD patients, three patients [15] and two patients [18]. In addition, there were two case reports [7,19] that consisted of a total number of three patients with IBD; these case reports were not included in the current assessment. The mean age of the patients from clinical studies was not reported, and for the case reports, patients had an age between 35 and 42 years old. In regards to the gender of patients, all clinical studies did not report; from case reports, there were two male patients and one female patient. 

### 3.3. Clinical Assessment of IBD

All included studies reported the diagnosis of CD; from the selected databases, no studies were found to report the treatment with dental implants in UC (Table 1). In these four clinical studies [15,16,17,18], the diagnosis of CD was taken from patients’ hospital records; no medical data in regards to CD assessment were presented. From the hospital records, smoking consumption, cardiovascular and gastric problems, osteoporosis, thyroid disorders, liver disease assessment, asthma, diabetes mellitus, presence of IBD, rheumatoid arthritis, oncological therapy, hysterectomy, and drugs intake were noted.

The patient from the case report of Cauble [19] presented a history of CD that deputed at the age of 13; then, at the age of 15, the patient presented with a lower grade of infection to bowel, which required the use of antibiotics for 5 years. During this period, complete bowel obstruction was presented twice; the second time, the patient required surgery for the resection of the bowel. The patient was placed on Prednisone and Infliximab for 3 years. After this treatment, the patient had another recurrence of bowel obstruction and was placed again on Prednisone and Infliximab. In addition, the patient reported that he was in a frequently debilitating pain, mostly in the stomach area.

Peron and collaborators [7] have reported two cases of CD. The first patient was diagnosed with CD (how the diagnosis of CD was performed is not available in this paper) and had ileocecal recectomy. As for drugs, he was indicated with Mesalazine, Prednisone, and Infliximab. As for general characteristics, this patient presented with diarrhea, abdominal pain, weight loss, and was smoking 10–15 cigarettes’/day since he was 18 years old. The second patient was diagnosed with CD (how the diagnosis of CD was performed is not available in this paper) and was prescribed with Mesalazine, Corticosteroids, and Infliximab. As general manifestations, this patient presented with diarrhea with abdominal pain, weight loss, and occasional episodes of fever (Table 2).

### 3.4. Clinical Assessment of Dental Implants

In the study of van Steenberghe and coworkers [15], patients received standard (pretapping the bone) and Mark II (self-tapping screw shaped implants, Nobel Biocare). Before implant insertion, a minimum bone height of 7 mm was required; each patient was classified according to the jaw bone resorption and bone quality of Lekholm and Zarb [20] quantified on CT scans. The total cohort of this study consisted of 399 patients, who received 1263 implants, from which, 27 implants were lost between 1- and 6-months post-insertion. When it comes to CD patients, 13 implants were placed in 3 patients. In 2 patients, 3 out of 10 implants showed early implant failure due to associated systemic diseases. In the last patients, zero out of three implants did not show implant failure. 

Alsaadi and coworkers [16] evaluated 1757 patients files who received 5759 implants (screw-shaped Branemark system, Nobel Biocare). The surgical protocol was the same as in the previous study [15]. In this study, the number of CD patients and number of implants inserted for these patients is missing. The authors have made a multi-variable GEE logistic regression between implant related, behavioral, local, and health factors, in which CD and osteoporosis were associated with implant failure. Moreover, they found a positive correlation between early implant failure associated with implant diameter, location, and smoking consumption. 

In another study of Alsaadi and coworkers [17], they evaluated the influence of local factors and systemic diseases on early implant failures. The surgical protocol was the same as in the previous studies [15,16]. A total number of 283 patients received 720 implants (MkIII TiUnitet implants, Nobel Biocare). Of these, 12 implants were inserted in CD patients (number of CD patients is missing). On GEE analysis, authors have found that CD was associated with early implant failure (1 out of 12 implants). 

The last retrospective study, published by Alsaadi and coworkers [18], aimed to assess the influence of local and systemic factors in implant failure over a period of 2 years. Authors analyzed 412 patients with 1514 implants inserted (Branemark system, Nobel Biocare). Of these, two patients were diagnosed with CD and received nine implants (of which, three implants failed to osseointegrate). 

The patient from Cauble’s case report [19] received dental extractions and after 6 months post-extraction, six dental implants (Straumann) on upper arch and four implants (Straumann) on lower arch plus bone grafts was performed (clinical details about the protocol of implant insertion was missing). The patient, after another 5 months post-implant insertion, received fixed hybrid prostheses in the upper arch; implant-supported porcelain fused-to-metal on the lower arch. He was submitted to a maintenance phase every 4 months. Other details from long-term follow-ups are missing (Table 2). 

Peron and coworkers [7] published a case series report in which the first patient received a dental extraction with immediate implant insertion, bone graft, and provisional crown (after 2 weeks, a final lithium di-silicate crown was inserted). The second patient, received the same surgical and prosthetic protocol as the first patient. Both patients were examined every 6 months and no crestal bone loss was present. After 1-year post-implant insertion and loading, the implants were esthetically and functionally stable.

### 3.5. Quality of the Included Studies

The results obtained according to STROBE criteria for the clinical studies are presented in Table 3. Overall, the quality of the included studies was good. As seen, two studies obtained a score of six points, and two studies obtained a score five points. All included studies did not report outcome measurements of dental implants failure in regards to the CD. Sample size of CD patients was reported in only two studies. 

## 4. Discussion

Several studies conducted over the years have reported a link between the presence of IBD and dental implants prognosis. The involved mechanisms of action describe the microbial imbalance theory, in which a higher content of peptides and a lower content of polysaccharides were observed in the intestinal microbiota. This leads to a microbial conversion in favor of anaerobic, Gram-negative microorganisms, with the impairment of the oral and periodontal tissues. Bacteria such as *Campylobacter rectus*, *Fusobacterium nucleatum,* or *Campylobacter concisus* were identified in relation to the two pathologies [21,22]. 

Other studies showed that the existence of antigen-bound antibodies, in the context of the IBD, can influence the process of osseointegration and the capacity of healing at the site of the implant [23]. Moreover, the elevated bone fracture risk, osteoporosis, and osteopenia were studied and confirmed to be present in chronic inflammatory disorders, due to the action of inflammation factors (such as IL-6, TNF, and IL-1β) [24]. In addition, aspects such as poor nutrition, hemorrhage, or infection should be taken into consideration when assessing a dental implants survival rate [25]. 

Another factor that should be taken into account is smoking. It has been stated that it may increase implant failure and the presence of oral infection, and may initiate oral cancer [26]. Several papers have mentioned that patients with CD have a higher incidence of complications and smoking cessation may provide up to a 65% reduction in the risk [27,28]. 

Scientific literature focuses not only on the effects of IBD on dental implants success, but also on the consequences produced by the specific medication. Biotechnological products, such as chimeric monoclonal antibodies or humanized monoclonal antibodies, can influence the process of dental surgery outcomes. There are three main categories that are used in the current treatment of inflammatory pathologies: modulators of lymphocytes B, inhibitors of TNF-a, and inhibitors of interleukin. All products are administered in the form of injections, but their direction of use differs, from the administration form (subcutaneous, intramuscular, or intravenous) to the distinct timelines (once a month, twice a month, twice a week, or weekly), according to the individual conditions [29]. Despite their therapeutic proven potential, the patient feedback to monoclonal antibodies differs from patient to patient. Furthermore, they may become gradually ineffective in a big number of cases, where subjects develop undesired immune responses [30]. Side effects of monoclonal antibodies were also identified, ranging from different cancer forms and infectious processes to tuberculosis reacutization episodes [31]. The most severe adverse effect in the oral cavity consists in the development of osteonecrosis of the jaw. Bevacizumab, sunitinib, and denosumab are the only products currently accepted by the FDA, as direct link to the osteonecrosis of the jaw. Nevertheless, studies reported cases of osteonecrosis in the presence of other monoclonal antibodies. Bevacizumab is an antibody directed against the growth factor related to the vascular endothelial growth, and is also responsible for the healing process, which intensifies the blood vessel permeability, and diminishes vasculogenesis and vasodilatation [32] Dental implants insertion is therefore considered dangerous in such situations. The mechanism of action of sunitinib is also linked to the process of healing and against the formation of new blood vessels [33], while denosumab attaches to the RANKL cytokine and despite its antiresorptive effect, can produce osteonecrosis. The variations in the treatment, such as periods of time between doses or the cumulative effect may represent factors in the appearance of the pathology [34]. 

A study reported, in 2018, the case of a 55-year-old woman, with five mandibular inserted implants, which developed infectious processes at all the five surgical sites, being under concomitant Adalimumab treatment, for UC. This complex case of infection, which was resolved by the removal of the dental implants and necrotic debris, was attributed to the TNF-a inhibitor treatment [35]. Another case report linked the osteonecrosis of the jaw, in a 49-year-old woman suffering from CD, to the Infliximab treatment. The treatment was performed by removal of the necrotic debris and resection of the affected bone, with integral recovery after one year and four months [36]. 

Medical products based on inhibitors of TNF-α are known to modify the ability of osseous healing after dental extraction procedures. Ferreira-Junior and coworkers in their experimental study on rats, have shown that TNF-α inhibitors are capable of altering bone repair capacity after dental extraction, mostly in the early period of healing [37]. In addition, expression of TNF-α, RANKL and OPG was lower in rats who received Infliximab. From this study, it can be taken away that Infliximab can determine the reduction in osteoclasts due to mononuclear precursors, which inhibits growth and groups. This may be translated also into the dental implant failure that has been seen into our included studies. 

Another interesting experimental study is from Kuchler and coworkers [38]. The authors assessed the influence of experimental colitis onto titanium miniscrews inserted into the tibia. They mentioned that intestinal inflammation in the early phase of bone healing in a rat colitis ruled out catabolic effects on bone turnover prior implant placement. The major finding mentioned was that experimental colitis did not influence bone healing around implants; the authors suggested that bone formation during remodeling and regeneration is independently controlled in this type of experimental colitis. 

Studies conducted over the years highlighted the association between IBD and bone deficit [39,40,41]. Among other autoimmune pathologies (e.g., lupus erythematosus, ankylosing spondylitis, or rheumatoid arthritis), IBD may interfere with the equilibrium of bone biology [39,40,41]. One factor may be represented by the activity of osteoblasts and osteocytes production and, on the other side, osteoclasts; these agents are well known to be a dynamic complex, in which balance is crucial for the normal function of bone metabolism [39]. This balance may be disturbed by the consumption of alcohol and smoking, lack of physical activity, low levels of vitamin D and calcium, use of drugs (e.g., cortisone), dietary deficiencies, or even the low production of gonadal hormones [39,40]. 

An essential role in bone metabolism alteration in patients with IBD is played by the production of proinflammatory cytokines. TGF, TNF-α, IL-1, IL-4, and IL-6 are currently known to interfere with bone density, by inhibiting bone formation and activating osseous resorption in both, CD and UC [40]. Nevertheless, there seems to be a distinction between the intrinsic mechanisms of CD, compared to UC, regarding the influence on the osseous activity. Ardizzone and coworkers showed that the long intake of corticosteroid treatment plays the most important role in UC bone loss, while, in CD, osseous deficiency seems to be attributed to the pathology per se [40]. Even more, Sylvester and coworkers highlighted that IL-6 is the sole cytokine with repeatedly increased serum values in CD patients, being constantly present in the circulation of individuals with coexisting osteoporosis [41]. 

These findings are of great importance in the decision of initiating a dental implant treatment, considering the changes that take place automatically in the structure of the alveolar bone. Qiao and coworkers, in a study on mice showed that accelerated alveolar bone loss was detected in the presence of IBD and was linked more to the pathology itself than to the inflammation of the periodontal tissue [42]. In addition, Vasovic and coworkers showed that these consequences are related to a low bone formation and a high bone turnover; these situations are exposing to an implant failure with a high rate of occurrence [6]. 

Considering these findings, there are authors who concluded that the advantages of dental implants are of great importance, improving the life status parameters and the function of the masticatory apparatus and can be offered as a solution to most of the patients, even to those with compromised background pathologies. The conditions that must be accomplished are the ones related to the input of prophylactic approaches, together with precise and strict recall sessions and the accurate monitorization of the case evolution [43]. 

Duttenhoefer and coworkers published a meta-analysis to investigate the influence of immunodeficiency on dental implant survival. Their analysis included four clinical studies and mentioned that there is a heterogeneity between IBD patients and dental implants with missing information. They showed a pooled OR of 8.12 with 95% CI (3.68, 17.92), concluding that implant failure is present in CD patients (this analysis included only two studies) [23]. The book chapter published by Javed and Romanos [7], started from the hypothesis that CD is associated with nutritional immune defects and these factors may increase early implant failure. All their included studies reported a prognosis of dental implant in CD patients and mentioned that the study population had additional various systemic disorders that may also have been a cause of implant failure. Authors gave a “D” level of evidence and concluded that there is insufficient evidence to sustain the stability of dental implants in CD. In the systematic review of Guobis and coworkers [44], they tried to assess the influence of different systemic disease and medications in implant therapy. Only one study was included [17] in this review; Guobis et al. stated that level of evidence is limited for CD and, in general, systemic disease may have potential to succeed in implant therapy. In another review published by Bornstein and coworkers [45], three studies with CD and implant therapy were included. Authors mentioned scarce literature in CD patients who received implant therapy and a clear conclusion on this pathology was not provided.

Taking all the risks into consideration, efforts must be undertaken in order to offer the appropriate implant therapy to patients who suffer from IBD (CD or UC). Concerning this affirmation, some researchers do not recommend dental implants to patients suffering from CD, due to the possibility of immune deficiency and malnutrition and, thus, to the possibility of implant failure [28]. However, other studies have proved that specific measures can be accomplished in order to offer implant therapy to patients suffering from IBD; these studies are only from case reports [7,19]. 

The major strengths of our review were the systematic search from the databases and the methodological assessment of the included studies. Nevertheless, the absence of clinical studies in which IBD and dental implants were used, makes these data a limitation of our study. Other limitations are represented by low available information about diagnosis of IBD, types of IBD treatment, number and types of dental implants used in IBD patients, and lack of follow-up after implant therapy. We started our review with the intent to obtain a meta-analysis; this was not possible due to the heterogeneity of the studies included, and, consequently, a statistical analysis could not be made.

## 5. Conclusions

The presence of clinical studies on the influence of IBD in implant therapy is limited. CD studies have stated that malnutrition, smoking, claustrophobia, or poor bone quality may be responsible for implant failure. For UC, no studies are available in the literature. When recommending an implant therapy to IBD patients, the multidisciplinary team should be aware of the side effects and caution measures should be taken according to the pathology of the patient. In order to draw a clear conclusion about the effects of IBD on implant therapy, more prospective clinical studies are needed.

## Figures and Tables

**Table 1:** Clinical studies included.

Author. Year. Country. Reference	Type of Study	Sample Size	Type of IBD	Dental Implant	Results	Conclusions
Van Steenberghe. 2002. Belgium [15]	Prospective	*n =* 3	Crohn’s disease	*n =* 13 (titanium implants, Nobel Biocare)	Implant failure: 2 patients showed early implant failure (3 out of 10 implants) implant failure due to other medical problems associated with Crohn’s disease: claustrophobia, poor bone quality, smoking (>10 cigarettes’/day) No implant failure: 1 patient did not show implant failure (0 out of 3 implants)	General factors increase implant failure rate. Crohn’s disease was associated with early implant failure.
Alsaadi. 2007. Belgium [16]	Retrospective	Not available	Crohn’s disease	Not available (titanium implants, Nobel Biocare)	In multivariable GEE logistic regression, Crohn’s disease was associated with implant failure (OR: 7.95; 95% CI (3.47, 18.24); *p* < 0.001) achieving statistical significance. Other details about implant failure in Crohn’s disease are not available.	Crohn’s disease significant related to implant failure. The use of dental implants should be considered when other prosthetic options are available in patients with systemic disease.
Alsaadi. 2008. Belgium [17]	Retrospective	Not available	Crohn’s disease	12 implants (MkIII TiUnitet implants, Nobel Biocare)	Implant failure (*n =* 1) No implant failure (*n =* 11) On GEE analysis (*p*-values: Fisher 0.21, GEE 0.02), Crohn’s disease was found to be associated with early implant failure. Other details about implant failure in Crohn’s disease are not available.	No definitive conclusion has been established. Crohn’s disease showed an early implant failure.
Alsaadi. 2008. Belgium [18]	Retrospective	*n =* 2	Crohn’s disease	9 implants (Screw-shaped Branemark system implants, or moderately rough very oxidized Ti-Unite surface, Nobel Biocare)	Implant failure (*n =* 3) No implant failure (*n =* 6) On GEE analysis (OR: 10.09; 95% CI (0.73,139.79); *p* = 0.09), no statistical significance was achieved. Other details about implant failure in Crohn’s disease are not available.	Crohn’s disease did not lead to late implant loss.

CI: confidence interval; GEE: generalized estimating equations; OR: odd ration.

**Table 2:** Case reports relevant for clinical practice.

Author. Year. Country. Reference	Sample Size	Type of IBD	Dental Diagnosis	Dental Treatment	Conclusions
Cauble. 2011. USA [19]	Male 42 years old	Crohn’s disease duration: 29 years (diagnosed at 13 years old)	Mild chronic periodontal disease Presence of dental decays on all remaining dental decays (except lower incisors); moderate wear due to attrition and erosion Remaining teeth structures were biomechanically compromised with restorative work Pulpal pathology Occlusal disfunction	Treatment of active decays Dental extraction + Bone grafts After 6 months of healing, 10 implants (Implant position upper arch: 3, 5, 6, 11, 12, 14; Implant position lower arch: 19, 21, 28, 30) (Straumann) were placed + provisional dentures After another 5 months, patient received: fixed hybrid prostheses in the upper arch; implant-supported porcelain fused-to-metal on the lower arch Maintenance every 4 months No report of implant failure.	The goal of the oral rehabilitation was to achieve a long-term success for chewing system and to obtain a pleasing esthetics. This patient was at biomechanical high risk, which was the determinant of his functional and esthetics problems.
Peron. 2015. Italy [7]	Patient 1 Male 35 years old	Crohn’s disease duration: 14 years (diagnosed at 21 years old)	Deep decay tooth 24: diagnosed as hopeless	Position tooth: 24, Dental extraction + dental implant (11.5 mm length, 4.7 mm diameter, Zimmer) + Particulate bone grafting material (Puros cancellous, Zimmer) + provisional crown After 2 weeks of healing, a final lithium di-silicate crown was inserted Patient was examined every 6 months with no crestal bone loss (after 13 months post-implant insertion) Patient presented another implant (11.5 mm length, 3.75 mm diameter, Tapered Screw Vent, Zimmer) for a period of 2 years with delayed loading, with no signs of peri-implantitis	Trabecular metal implant showed osseointegration capacity and remained stable in patients in Crohn’s disease patients.
	Patient 2 Female 36 years old	Crohn’s disease duration: 11 years (diagnosed at 25 years old)	Deep decay tooth 14: diagnosed as hopeless	Surgical and prosthetic protocol was the same as in Patient 1 After 1 year of loading, implant did not show any complications.	

**Table 3:** Risk of bias assessment.

Reference	Study Design	Participants	Sample Size	Variable Description	Potential Confounders	Outcome Measurements	Statistical Analysis	Total Score
van Steenberghe [15]	1	1	1	1	1	0	1	6
Alsaadi [16]	1	1	0	1	1	0	1	5
Alsaadi [17]	1	1	0	1	1	0	1	5
Alsaadi [18]	1	1	1	1	1	0	1	6

## Data Availability

Not applicable.
